# Certainty equivalence-based robust sliding mode control strategy and its application to uncertain PMSG-WECS

**DOI:** 10.1371/journal.pone.0281116

**Published:** 2023-02-27

**Authors:** Annas Chand, Qudrat Khan, Waqar Alam, Laiq Khan, Jamshed Iqbal

**Affiliations:** 1 Department of Electrical and Computer Engineering, COMSATS University Islamabad, Abbottabad Campus, Abbottabad, Pakistan; 2 Centre for Advanced Studies in Telecommunications (CAST), COMSATS University Islamabad, Islamabad, Pakistan; 3 Department of Electrical and Computer Engineering, COMSATS University Islamabad, Islamabad, Pakistan; 4 Department of Computer Science and Technology, Faculty of Science and Engineering, University of Hull, Hull, United Kingdom; J.C. Bose University of Science and Technology, YMCA, INDIA, INDIA

## Abstract

This work focuses on maximum power extraction via certainty equivalence-based robust sliding mode control protocols for an uncertain Permanent Magnet Synchronous Generator-based Wind Energy Conversion System (PMSG-WECS). The considered system is subjected to both structured and unstructured disturbances, which may occur through the input channel. Initially, the PMSG-WECS system is transformed into a Bronwsky form, i.e., controllable canonical form, which is composed of both internal and visible dynamics. The internal dynamics are proved stable, i.e., the system is in the minimum phase. However, the control of visible dynamics, to track the desired trajectory, is the main concern. To carry out this task, the certainty equivalence-based control strategies, i.e., conventional sliding mode control, terminal sliding mode control and integral sliding mode control are designed. Consequently, a chattering phenomenon is suppressed by the employment of equivalent estimated disturbances, which also enhance the robustness of the proposed control strategies. Eventually, a comprehensive stability analysis of the proposed control techniques is presented. All the theoretical claims are verified via computer simulations, which are performed in MATLAB/Simulink.

## 1 Introduction

In the current era, due to the depletion of fossil fuels and their environmental impacts, researchers have focused on renewable energy resources (RES). In various renewable energy resources, energy harnesses from wind are getting much importance due to their sustainable and environment-friendly nature [1–3]. The system used for the said purpose is Permanent Magnet Synchronous Generator-based Wind Energy Conversion System (PMSG-WECS).

WECS is either autonomous or grid-connected [4]. However, depending upon the wind speed that is generated using the anemometer data, WECS can be operated within three different regimes, i.e., no load, partial load and constant load [5]. For partial load, the efficiency of the WECS is more crucial. Thus, to maximise the WECS efficiency, in a partial load scenario, the Maximum Power Point Tracking (MPPT) method has been presented. The controller in MPPT acts as a backbone in the operation of MPPT, which captures maximum energy from wind. While its control functioning is directly related to the operating characteristics, economic effective generation and equipment security stability. So far, various kinds of control strategies are proposed for MPPT design, which include [6, 7], PID control, model predictive control [8], neuro-fuzzy control [9, 10], adaptive backstepping control [11, 12], sliding mode control scheme [13–15] and an integral-based terminal sliding mode control strategy [16]. Moreover, interconnection and damping assignment-based control schemes are presented in [17, 18]. The aforementioned control strategies are synthesized for the WECs to get maximum efficiency, i.e., MPPT, while taking into account the PMSG’s entire dynamic. However, each strategy has its own merits and demerits. Recently, a continuous switching-based sliding mode control scheme is presented in [19]. The proposed control strategy, initially, regulates the grid and the generator side converter to track the desired reference speed. Secondly, it alleviates the chattering issue associated with the conventional sliding mode control scheme.

In distant and harsh circumstances, during a long-term ongoing operation, partial failure of electro-mechanical parts, i.e., gearbox, motor, alternator and power electronic converter, is inevitable [20–23]. These partial faults may lead to poor performance of the actuators, which can result in performance degradation and efficient operation of PMSG-WECS. Therefore, keeping in view the safety, high reliability and long life of WECS, the research community have focused on robust methods, for the control design, to ensure excellent working in uncertain situations.

Robust control strategy ensures the system stability and specific performance not only in the nominal scenarios but also in case of external uncertainties [24–28]. It either counteracts the fault with efficient robustness by using a fixed gain controller or implements a fast dynamic compensation control input. Regarding WECS, the robust control techniques used in the literature are signal-based approach [29], hardware redundancy method [30], data-driven techniques [31], Barrier function-based adaptive non-singular sliding mode control approach [32], fractional-order sliding mode control technique [33], convolution neural network method [34], fuzzy method [35], global sliding mode control approach [36] and sliding mode observer method [37, 38]. Using these techniques, the information on the uncertainty, that occurred in PMSG-WECS, is obtained, which is then adjusted by the designed control law.

It is quite evident that, before designing the controller, the robust control methods require the designed engineers to forecast the bounds of the expected faults/uncertainty. During the faulty condition, a robust control scheme works according to the dynamics of the system, which need to be adjusted according to the specific fault dynamics. Its benefit is the simplicity of the control law, which can ensure a system’s stability and attain predetermined efficiency irrespective of a fault. During severe uncertain situations, the controller must be able to find out the exact gain parameters. As the complexity of the system increases, the design process of the controller becomes more difficult. In [39], a certainty equivalence-based super-twisting algorithm (CESTA) is implemented on a diesel engine for the diagnosis of match uncertainties and later it is counteracted. In [40], the certainty equivalence-based integral sliding mode control (CEISMC) technique is being utilized to diagnose and mitigate the actuator fault for the diesel engine.

It is worth mentioning that our contribution to this work is in three folds. Firstly, the dynamics of the PMSG-WECS are modelled via Park transformation and later transformed into a controllable canonical form, which is a feasible structure and assists us in the design of the control strategy. Secondly, a certainty equivalence-based conventional sliding mode control (CSCMC), terminal sliding mode control (TSMC) and integral sliding mode control are designed. The stability analysis of the designed control strategies is provided in a comprehensive manner. Moreover, the effectiveness of the designed control strategies is demonstrated in MATLAB/Simulink. In addition, the comparative analysis of the aforementioned control schemes is carried out as a third contribution.

This paper is organized as follows; Standalone PMSG-WECS modelling is presented in section 2. Section 3 presents the controllable canonical form and the investigation of zero dynamics in a nonlinear PMSG-WECS system. The design of CECSMC, CETSMC and CEISMC along with Lyapunov stability analysis are outlined in section 4. In section 5, simulations and discussion are presented whereas section 6 concludes the paper. In last, a declaration of conflicting interests is provided in section 7.

## 2 Mathematical modelling of standalone PMSG-WECS

This section is composed of two subsystems, i.e., rotor blade modelling and PMSG modelling, which is further connected to a load. Both are discussed comprehensively in the forthcoming subsections.

### 2.1 Aerodynamic modelling of wind turbine

As a result of fast-moving wind, which struck against the wind turbine blades, the linear wind energy is transformed into mechanical energy. The mechanical power generated via this phenomenon is represented as follows [4]

$$\begin{array}{c} P_{m}=\frac{1}{2}\rho \pi {R_{t}}^{2}{v_{w}}^{3}C_{p}\left(\lambda ,\theta \right), \end{array}$$ (1)

where *P*_*m*_ is the mechanical power, *R*_*t*_ is radius of blades of the wind turbine, *ρ* is density of the air, *v*_*w*_ is the speed of the wind, λ is the tip speed ratio (TSR), *θ* is the pitch angle, *C*_*p*_ is the power coefficient, which depends on the λ and *θ*.

**Assumption 1** *It is assumed that the pitch angle is constant and is kept at zero i.e*., (*θ* = 0).

**Fig 1:**
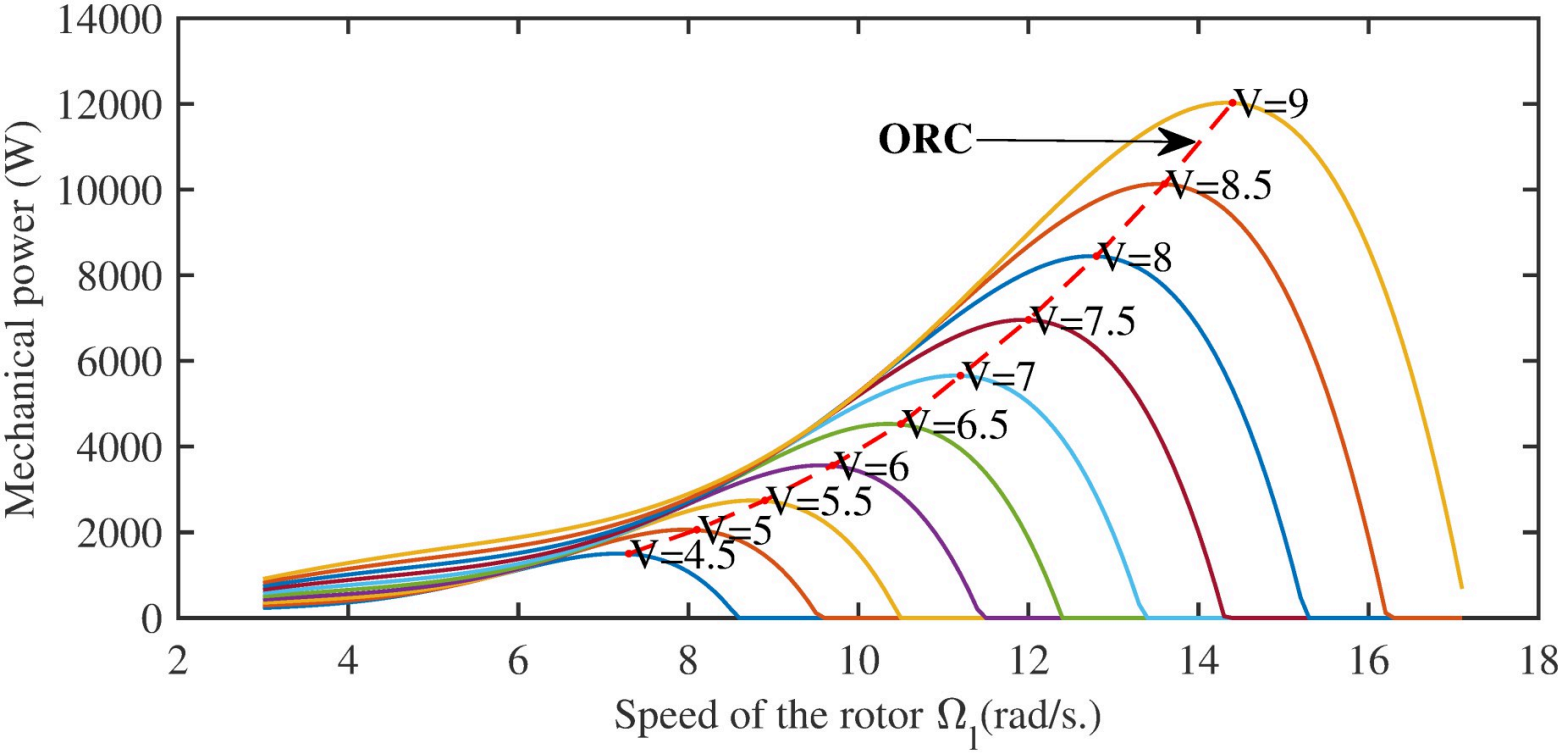
Mechanical power versus rotor speed.

A TSR is the ratio of the blade tip speed to the wind speed. The detailed expression of the TSR appears as follows

$$\begin{array}{c} \lambda =\frac{\Omega_{l}R_{t}}{v_{w}}, \end{array}$$ (2)

where Ω_*l*_ is the rotational speed of the wind turbine blades at a low-speed shaft. As clearly seen in Fig 1, the mechanical output power of the wind turbine increases according to the wind speed. For every wind speed curve, there is a specific peak power point. By joining all these peak power points, a curve is formed which is known as optimal regime characteristics (ORC) [5]. For every wind speed *v*_*w*_, there is a specific generator speed at which the power coefficient *C*_*p*_ reaches its maximum value, i.e., *C*_*pmax*_, whenever λ becomes λ_*opt*_. So, in order to harvest maximum power from wind, the TSR should be kept at its optimal value, λ_*opt*_, in such a way that the shaft speed Ω_*h*_ exactly tracks the reference speed, Ω_*ref*_, which is defined as follows

$$\begin{array}{c} \Omega_{ref}=\frac{\lambda_{opt}v_{w}}{R_{t}} \end{array}$$ (3)

The rotor power of PMSG is given as follows

$$\begin{array}{c} P_{t}=\Gamma_{t}\Omega_{l} \end{array}$$ (4)

The aerodynamic torque is given by

$$\begin{array}{c} \Gamma_{t}=\frac{1}{2}\pi {R_{t}}^{3}{v_{w}}^{2}C_{q}\left(\lambda \right), \end{array}$$ (5)

where *C*_*q*_(λ) is the coefficient of torque, which can be expressed as follows

$$\begin{array}{c} C_{q}\left(\lambda \right)=\frac{C_{p}\left(\lambda \right)}{\lambda }, \end{array}$$ (6)

where *C*_*q*_(λ), *C*_*p*_(λ) and λ_*opt*_ are the designed parameters, which are provided by the manufacturer of the wind turbine. Now, all the necessary components of the wind turbine are modelled. In the subsequent subsection, the PMSG modelling is displayed.

### 2.2 Modelling of the Permanent Magnet Synchronous Generator (PMSG)

As the considered PMSG is a standalone system, the power generated is pre-processed for the sake of compatibility before it is stored in the battery banks for later use. The *dq*-model of the PMSG, while ignoring the zero dynamics, is given by

$$\begin{array}{c} \frac{d}{dt}i_{d}=\frac{-Ri_{d}+L_{q}i_{q}+u_{d}}{L_{d}}\\ \frac{d}{dt}i_{q}=\frac{-Ri_{q}-(L_{d}i_{d}+\phi_{m})+u_{q}}{L_{q}}\\ \frac{d}{dt}\Omega_{h}=\frac{1}{J_{h}}\left(\Gamma_{t}-\Gamma_{em}\right)=\frac{\Gamma_{t}}{J_{h}}-\frac{p\left(\phi_{d}i_{q}-\phi_{q}i_{d}\right)}{\Gamma_{t}} \end{array}\}$$ (7)

where *J*_*h*_ is the moment of inertia of the generator shaft. *u*_*d*_, *u*_*q*_, Ω_*h*_, Γ_*em*_, *i*_*d*_, *i*_*q*_, *L*_*d*_, and *L*_*q*_, *R*, *p*, Φ_*d*_ = *L*_*d*_*i*_*d*_ + Φ_*m*_, Φ_*q*_ = *L*_*q*_*i*_*q*_ and Φ_*m*_ are the voltages of the DQ-axis, PMSG speed, the electromagnetic torque, DQ-axis current and rotor inductance, the resistance of the stator, pole pair, DQ and the linkage flux, respectively. The under study system is non-salient synchronous generator so *L*_*d*_ = *L*_*q*_ = *L*. The torque of the generator can be written as

$$\begin{array}{cc} \Gamma_{em} & =p\Phi_{m}i_{q} \end{array}$$ (8)

Consider the following assumptions while modeling PMSG.

**Assumption 2** *Stator winding should have sinusoidal distribution with having an electrical and magnetic symmetry. In addition, the iron losses are not considered*.

**Remark 1** *The dynamics of the electronic circuit are neglected, due to its faster nature as compared to the dynamics of the PMSG-WECS*.

The nonlinear dynamics of the SISO PMSG-WECS presented in (7) and (8) can be formulated as

$$\begin{array}{c} {\dot{x}}_{1}=\frac{-R_{s}x_{1}+p\left(L_{q}-L_{ch}\right)x_{2}x_{3}-R_{ini}x_{1}}{\left(L_{d}+L_{ch}\right)}\\ {\dot{x}}_{2}=\frac{-R_{s}x_{2}-p\left(L_{d}+L_{ch}\right)x_{1}x_{3}-R_{ini}x_{2}}{\left(L_{q}+L_{ch}\right)}+p\Phi_{m}x_{3}\\ {\dot{x}}_{3}=\frac{\frac{d_{1}v_{w}^{2}}{i}+\frac{d_{2}v_{w}x_{3}}{i^{2}}+\frac{d_{3}x_{3}^{2}}{i^{3}}-p\Phi_{m}x_{2}}{J_{h}} \end{array}\}$$ (9)

The matrix [*x*_1_ *x*_2_ *x*_3_]^*T*^ = [*i*_*d*_ *i*_*q*_ Ω_*h*_]^*T*^ ∈ ℜ^3^, indicates the states vector of the PMSG model. Ω_*h*_ = Ω_*l*_ × *i* is the generator speed, *L*_*ch*_ and *R*_*ch*_ are the inductance and resistance of the load, respectively. *i* is the mechanical transmission ratio, *R*_*ch*_ is considered as a control input. *L*_*d*_ and *L*_*q*_ represent the stator’s dq-axes inductance while *i*_*d*_ and *i*_*q*_ stand for the dq-axes stator’s current, respectively.

## 3 Controllable canonical form

The dynamics of the PMSG-WECS model, in generic form, can be expressed as

$$\begin{array}{c} \dot{x}=f\left(x\right)+g(x)u\\ y=h\left(x\right) \end{array}\}$$ (10)

The variable *x* ∈ ℜ^*n*^ represents the state vector. The control input is described by *u* ∈ ℜ^*m*^, while *f*(*x*) and *g*(*x*) are the smooth nonlinear vectors which are expressed as

$$f\left(x\right)=[\begin{array}{c} \frac{-R_{s}x_{1}+p\left(L_{q}-L_{ch}\right)x_{2}x_{3}}{\left(L_{d}+L_{ch}\right)}\\ \frac{-R_{s}x_{2}-p\left(L_{d}+L_{ch}\right)x_{1}x_{3}}{\left(L_{q}+L_{ch}\right)}+p\Phi_{m}x_{3}\\ \frac{\frac{d_{1}v_{w}^{2}}{i}+\frac{d_{2}v_{w}x_{3}}{i^{2}}+\frac{d_{3}x_{3}^{2}}{i^{3}}-p\Phi_{m}x_{2}}{J_{h}} \end{array}],$$

$$g\left(x\right)=[\begin{array}{c} \frac{-x_{1}}{\left(L_{d}+L_{ch}\right)}\\ \frac{-x_{2}}{L_{q}+L_{ch}}\\ 0 \end{array}]$$

where

$$u=R_{ch}$$

The output is represented by *y* = *h*(*x*) = *x*_3_ = Ω_*h*_, which describes the angular speed of the rotor shaft. As the objective is to control the output, thus, (9) is transformed into a controllable canonical structure, i.e., input-output form, via the following transformation.

$$\begin{array}{c} z_{1}=y=h\left(x\right)=x_{3}=\Omega_{h}\\ z_{2}=L_{f}h(x)=\frac{\partial h(x)}{\partial x}.f(x)\\ z_{3}=L_{f}^{2}h(x)=\frac{x_{1}}{x_{2}} \end{array}\}$$ (11)

It is quite obvious that the relative degree ‘*r*’ of the considered system is one less than the system order, i.e., (*r* < *n*) as *n* = 3. So the input-output form can be expressed as

$$\begin{array}{c} {\dot{z}}_{1}=z_{2}\\ {\dot{z}}_{2}=L_{f}^{2}h(x)+L_{g}L_{f}h(x)u \end{array}\}$$ (12)

$$\begin{array}{c} {\dot{z}}_{3}=-\frac{m_{4}}{m_{1}}(\frac{k_{1}z_{3}m_{1}}{m_{4}}+\frac{k_{2}z_{1}m_{1}}{m_{4}}+\frac{k_{3}z_{3}m_{1}u}{m_{4}})+(\frac{z_{3}m_{1}}{m_{4}})(\frac{m_{4}^{2}}{m_{1}^{2}})(-\frac{l_{1}m_{1}}{m_{4}}\frac{l_{2}m_{1}z_{3}z_{1}}{m_{4}}-l_{3}z_{1}+\frac{l_{4}m_{1}u}{m_{4}}) \end{array}$$ (13)

The system under consideration, when converted to an input-output form, contains an internal dynamic, i.e., zero-dynamic. The stability of the zero-dynamics is quite crucial to be discussed.

**Remark 2** *The* Eq (13) *remains no more dependent on the control input. This system is affected only by the control-driven states i.e*., *z*_1_ *and* *z*_3_. *Its zero dynamics will be discussed in subsequent paragraphs*.

The typical plant parameters and the derived parameters are given in Tables 1–3. Note that the nonlinear system (12), is driven by the applied control input u whereas system (13), with states (*z*_1_, *z*_3_), represents the internal dynamics whose stability, zero-dynamics stability, will be discussed in the following subsection.

**Table 1:** Gains used in model.

Symbol	Value	Symbol	Value
*m*_1_	27.14709	*m*_2_	-0.94867
*m*_3_	8.22639	*h*_1_	-27.14709
*h*_2_	3	*h*_3_	10.81441
*h*_4_	8.22639	*K*_1_	188.3636
*K*_2_	-2.5234	*K*_3_	-0.009587
*K*_4_	1.3146	*d*_1_	3.8841
*d*_2_	-0.3604	*d*_3_	-0.009587

**Table 2:** Parameters of wind turbine.

Nomenclature	Symbol	Value
Gears ratio	*i*	7
Air density	*ρ*	1.25 *kg*/*m*^3^
Radius of Blade	*R*_*t*_	2.5 *m*
HSS Inertia	*J*_*h*_	0.0552 *kg*.*m*^2^
Maximum power coefficient	*C*_*pmax*_	0.47
Optimal tip speed ratio	λ_*opt*_	7

**Table 3:** Parameters of PMSG.

Nomenclature	Symbol	Value
Stator’s resistance	*R*	3.3 *ohm*
Pole pair	*p*	3
Flux	*ϕ*_*m*_	0.4382 *Wb*
Equivalent resistance of chopper	*R*_*ch*_	80 *ohm*
Direct-axis inductance	*L*_*d*_	41.56 *mH*
Quadrature-axis inductance	*L*_*q*_	41.56 *mH*
Chopper inductance	*L*_*ch*_	0.08 *H*

### 3.1 Stability analysis of the zero-dynamics

The dynamics of the nonlinear system are subdivided into two subsystems, i.e., visible dynamics system and internal dynamics system [41]. To find out the zero dynamics, choosing *z*_1_ = *z*_2_ = 0 in (13) and simplifying it, one comes with

$$\begin{array}{cc} {\dot{z}}_{3} & =-z_{3}[-h_{1}+m_{1}-m_{2}\frac{K_{1}}{K_{4}}] \end{array}$$ (14)

Owing to Table 1, the constant $-h_{1}+m_{1}-m_{2}\frac{K_{1}}{K_{4}}=\tau$ is positive with numerical value 190.21.

$$\begin{array}{cc} \dot{z_{3}} & =-\tau z_{3} \end{array}$$ (15)

This equation shows that the zero dynamics are strongly asymptotically stable. Thus, the system under study is the minimum phase.

**Remark 3** *The system developed in* (12) *and* (13), *in practice, experiences a different kinds of disturbances*.

Hence, the system (12) and (13), in practical form can be described as follows

$$\begin{array}{c} {\dot{z}}_{1}=z_{2}\\ {\dot{z}}_{2}={L_{f}}^{2}h(x)+L_{g}L_{f}h(x)\left(u(1-G)+F(x,t)\right)\\ \dot{z_{3}}=-\tau z_{3} \end{array}\}$$ (16)

where *G* represents the health of the input channel. If *G* = 0, it means that the system’s input channel is healthy and 0 < *G* < 1 indicates the unhealthy nature of the input channel. In addition, *F*(*x*, *t*) represents the uncertainties about which the following assumption is made.

**Assumption 3** *Assume that the uncertain terms can be subdivided into structured and unstructured terms, i.e*.,

$$\begin{array}{c} F\left(x,t\right)=f_{st}\left(x\right)+f_{un}\left(x,t\right) \end{array}$$ (17)

*The bound of the unstructured faults/uncertainties are defined as*

$$\begin{array}{c} |f_{un}\left(x,t\right)|\leq \vartheta , \end{array}$$ (18)

*where ϑ is a positive constant*.

$$\begin{array}{c} f_{st}\left(x\right)=\Delta \Psi \left(x\right) \end{array}$$ (19)

The partially known structured uncertainty in (19) can be expressed as the product of an unknown constant parameter Δ and a known base function Ψ(*x*). Δ can be any parametric change, which occurs in the internal parameters of the wind system. Structured faults/uncertainties may be unknown plant parameters like resistance values or friction coefficients whereas unstructured faults may represent external disturbances. The system (16) represents a complete model of WECS-PMSG. In the next section, the control design will be focused on.

## 4 Certainty equivalence-based sliding mode control strategy

In this section, a synthetic structure of sliding modes and adaptive control is proposed. Conventional sliding mode control (SMC) scheme claims invariance property subjected to the design of the sliding surface and the gains of the discontinuous part. However, it may result in high chattering phenomena which could be dangerous for the actuators and the system’s health. Therefore, a certainty equivalence-based sliding mode control protocol is proposed. The beauty of this strategy is that the robustness of the controller remains higher and the chattering is eliminated or suppressed, which is not possible in the conventional SMC. The design is outlined in the following subsection.

### 4.1 Certainty equivalence-based conventional SMC design

It is quite worth mentioning that the main task of the current work is the extraction of maximum power from the WECS, which can be done by following a reference signal. Thus, reference tracking is the ultimate objective.

**Assumption 4** *It is assumed that the reference speed is of class* *C*^1^.

Now, by defining the error as follows

$$\begin{array}{c} e=z_{1}-z_{1ref},\;\;\;\;\;\dot{e}={\dot{z}}_{1}-{\dot{z}}_{1ref} \end{array}$$ (20)

To pursue the design, a sliding surface/manifold, in terms of error variable, is defined as follows

$$\begin{array}{c} s=\dot{e}+c_{1}e, \end{array}$$ (21)

where *c*_1_ is a positive constant. The time derivative of (21) along (20) and (16), becomes

$$\begin{array}{c} \dot{s}={L_{f}}^{2}h(x)+L_{g}L_{f}h(x)(u+F(x,t))-{\ddot{z}}_{1ref}+c_{1}\dot{e} \end{array}$$ (22)

Substituting the match faults/uncertainties from (18) and (19) in (22), the following expression is obtained

$$\begin{array}{c} \dot{s}={L_{f}}^{2}h(x)+L_{g}L_{f}h(x)(u+f_{st}\left(x\right)+f_{un}\left(x,t\right))-{\ddot{z}}_{1ref}+c_{1}\dot{e} \end{array}$$ (23)

The final control law, *u* composed of an equivalent *u*_*eq*_ and discontinuous *u*_*d*_ control laws, which can be written as

$$\begin{array}{c} u=u_{o}+u_{eq}+u_{d} \end{array}$$ (24)

Invoking (24) in (23), one gets

$$\begin{array}{c} \dot{s}={L_{f}}^{2}h(x)+L_{g}L_{f}h(x)(u_{o}+u_{eq}+u_{d}+f_{st}\left(x\right)+f_{un}\left(x,t\right))-{\ddot{z}}_{1ref}+c_{1}\dot{e} \end{array}$$ (25)

To calculate the equivalent control input, the uncertain terms and $\dot{s}$ must be equal to zero, which gives us the following expression.

$$\begin{array}{c} u_{eq}=-\frac{1}{L_{g}L_{f}h(x)}({L_{f}}^{2}h(x)-{\ddot{z}}_{1ref}+c_{1}\dot{e}) \end{array}$$ (26)

In order to mitigate the effects of the structured uncertainties, an equivalent cancellation law *u*_*o*_ is proposed as follows

$$\begin{array}{c} u_{o}=-\hat{\Delta }\Psi (x) \end{array}$$ (27)

where $\hat{\Delta }$ represents the estimated value of the unknown parameter. The discontinuous control law *u*_*d*_ is designed as follows

$$\begin{array}{c} u_{d}=-\frac{1}{L_{g}L_{f}h(x)}(M_{1}s+M_{2}sign\left(s\right)), \end{array}$$ (28)

where *M*_1_ and *M*_2_ are the positive gains. The obtained control law is as follows

$$\begin{array}{c} u=-\hat{\Delta }\Psi \left(x\right)-\frac{1}{L_{g}L_{f}h(x)}({L_{f}}^{2}h(x)-{\ddot{z}}_{1ref}+c_{1}\dot{e})-\frac{1}{L_{g}L_{f}h(x)}(M_{1}s+M_{2}sign\left(s\right)) \end{array}$$ (29)

The aforementioned final control law enforces the sliding mode along the sliding surface given in (21). To prove the closed loop stability, i.e., sliding mode enforcement, consider a Lyapunov candidate function (LCF) as follows

$$\begin{array}{c} V=\frac{1}{2}s^{2}+\frac{1}{2\gamma }{\tilde{\Delta }}^{2}, \end{array}$$ (30)

where *γ* > 0 and $\tilde{\Delta }$ = Δ − $\hat{\Delta }$ is the error between the actual and estimated parameter. Now, consider the time derivative of (30), one has

$$\begin{array}{c} \dot{V}=s\dot{s}+\frac{1}{\gamma }\tilde{\Delta }\left(\dot{\Delta }-\dot{\hat{\Delta }}\right) \end{array}$$ (31)

Substituting (23) into (31), we get

$$\begin{array}{c} \dot{V}=s({L_{f}}^{2}h(x)+L_{g}L_{f}h(x)\left(u+f_{st}\left(x\right)+f_{un}\left(x,t\right)\right)-{\ddot{z}}_{1ref}+c_{1}\dot{e})+\frac{1}{\gamma }\tilde{\Delta }\left(0-\dot{\hat{\Delta }}\right) \end{array}$$ (32)

Using Assumption 3, the following expression is obtained

$$\begin{array}{c} \dot{V}=s({L_{f}}^{2}h(x)+L_{g}L_{f}h(x)u+L_{g}L_{f}h(x)\Delta \Psi (x)+L_{g}L_{f}h(x)f_{un}\left(x,t\right)\\ -{\ddot{z}}_{1ref}+c_{1}\dot{e})+\frac{1}{\gamma }\tilde{\Delta }\left(0-\dot{\hat{\Delta }}\right) \end{array}$$ (33)

Using values of *u*_*eq*_, *u*_*o*_ and *u*_*d*_, one gets the following expression

$$\begin{array}{c} \begin{array}{c} \dot{V}=s({L_{f}}^{2}h(x)+L_{g}L_{f}h(x)u_{eq}+L_{g}L_{f}h(x)u_{d}-L_{g}L_{f}h(x)\hat{\Delta }\Psi \left(x\right)+L_{g}L_{f}h(x)\Delta \Psi (x)+L_{g}L_{f}h(x)f_{un}\left(x,t\right) \end{array} \end{array}$$

$$\begin{array}{c} \dot{V}=s(-M_{1}s-M_{2}sign\left(s\right)+L_{g}L_{f}h(x)f_{un}\left(x,t\right))+sL_{g}L_{f}h(x)\Psi (x)\left(\Delta -\hat{\Delta }\right)-\frac{1}{\gamma }\tilde{\Delta }\dot{\hat{\Delta }} \end{array}$$

$$\begin{array}{c} \dot{V}\leq -M_{1}s^{2}-\left|s\right|(M_{2}-\Gamma_{m}\vartheta )+\tilde{\Delta }(sL_{g}L_{f}h(x)\Psi (x)-\frac{1}{\gamma }\dot{\hat{\Delta }}) \end{array}$$ (34)

Now, choosing $\dot{\hat{\Delta }}=\gamma sL_{g}L_{f}h(x)\Psi \left(x\right)$, the derivative of LCF can be written as

$$\begin{array}{c} \dot{V}\leq -M_{1}s^{2}-\left|s\right|(M_{2}-\Gamma_{m}\vartheta )+0 \end{array}$$ (35)

Now, choose the following expression

$$\begin{array}{c} M_{2}-\Gamma_{m}\vartheta \geq \eta >0, \end{array}$$ (36)

one gets

$$\begin{array}{c} \dot{V}\leq -M_{1}s^{2}-\eta \left|s\right| \end{array}$$ (37)

The inequality 37 proves the negative definiteness of the LCF. Hence, it is confirmed that sliding mode enforcement is achieved in finite time, i.e., *s* → 0, subjected to the conditions, i.e., *M*_2_ ≥ *η* + Γ_*m*_*ϑ* and $\dot{\hat{\Delta }}=\gamma sL_{g}L_{f}h(x)\Psi \left(x\right)$. This proves the theorem.

**Remark 4** *The value of the adaptation gain parameter* $\hat{\Delta }$ *will remain close to zero where there is no structured uncertainty in the framework. However, when some fault affects the framework, the value of the adaptation gain parameter increases according to the magnitude of the fault. A non-zero value of the adaptation parameter indicates the presence of disturbances*.

The above-mentioned strategy is developed with terminal SMC which is discussed in the subsequent subsection.

### 4.2 Certainty equivalence-based terminal sliding mode control strategy

To pursue the design of a certainty equivalence-based TSMC scheme, consider the tracking error and its time derivative, the terminal sliding manifold [42] is defined as

$$\begin{array}{c} s=\dot{e}+\alpha e+\beta e^{\frac{p}{q}}, \end{array}$$ (38)

where *α*> *β* > 0, *p* and *q* are odd positive numbers such that $0\;<\frac{p}{q}<\;1$.

**Remark 5** *The difference between* (21) *and* (38) *is simply the addition of the new term on the right-hand side of* (38). *The beauty of this manifold is that, as a sliding mode is enforced, the error dynamics converge to zero in finite time instead of asymptotic convergence which results in high precision as compared to CSMC*.

Taking the time derivative of (38) along (20) and (16), the following expression is obtained.

$$\begin{array}{c} \dot{s}={L_{f}}^{2}h(x)+L_{g}L_{f}h(x)(u+F(x,t))-{\ddot{z}}_{1ref}+\alpha \dot{e}+\beta \frac{p}{q}e^{\left(\frac{p}{q}-1\right)}\dot{e} \end{array}$$ (39)

Substituting (18) and (19) into (39), it yields

$$\begin{array}{c} \dot{s}={L_{f}}^{2}h(x)+L_{g}L_{f}h(x)(u+f_{st}\left(x\right)+f_{un}\left(x,t\right))-{\ddot{z}}_{1ref}+\alpha \dot{e}+\beta \frac{p}{q}e^{\left(\frac{p}{q}-1\right)}\dot{e} \end{array}$$ (40)

The overall control law appears as follows

$$\begin{array}{c} u=u_{o}+u_{eq}+u_{d}, \end{array}$$ (41)

where known terms will be canceled by *u*_*eq*_ and matched faults will be handled by *u*_*d*_ and *u*_*o*_. Invoking (41) in (40), one gets the following expression.

$$\begin{array}{c} \dot{s}={L_{f}}^{2}h(x)+L_{g}L_{f}h(x)(u_{o}+u_{eq}+u_{d}+f_{st}\left(x\right)+f_{un}\left(x,t\right))-{\ddot{z}}_{1ref}+\alpha \dot{e}+\beta \frac{p}{q}e^{\left(\frac{p}{q}-1\right)}\dot{e} \end{array}$$ (42)

Ignoring the disturbances, *u*_*o*_ and *u*_*d*_ in (42), one gets

$$\begin{array}{c} u_{eq}=-\frac{1}{L_{g}L_{f}h(x)}({L_{f}}^{2}h(x)-{\ddot{z}}_{1ref}+\alpha \dot{e}+\beta \frac{p}{q}e^{\left(\frac{p}{q}-1\right)}\dot{e}) \end{array}$$ (43)

In order to cancel the effects of structured uncertainties, an equivalent cancellation law *u*_*o*_ is proposed as follows

$$\begin{array}{c} u_{o}=-\hat{\Delta }\Psi (x), \end{array}$$ (44)

where $\hat{\Delta }$ represents the estimated value of the unknown parameter. The discontinuous control law *u*_*d*_ appears as follows

$$\begin{array}{c} u_{d}=-\frac{1}{L_{g}L_{f}h(x)}(M_{1}s+M_{2}sign\left(s\right)), \end{array}$$ (45)

The overall control law can be written as

$$\begin{array}{c} u=-\hat{\Delta }\Psi \left(x\right)-\frac{1}{L_{g}L_{f}h(x)}({L_{f}}^{2}h(x)-{\ddot{z}}_{1ref}+\alpha \dot{e}+\beta \frac{p}{q}e^{\left(\frac{p}{q}-1\right)}\dot{e})-\frac{1}{L_{g}L_{f}h(x)}(M_{1}s\\ +M_{2}sign\left(s\right)) \end{array}$$ (46)

This control law enforces the sliding mode along the sliding surface. Consequently, the system’s output tracks the desired reference in a finite time. The stability analysis of the current control scheme and the aforementioned control strategy is quite similar. The only difference is the few additional terms in the sliding manifold and the equivalent control law. Thus, the details are avoided here. Again, the same strategy is developed with integral SMC which is presented below.

### 4.3 Certainty equivalence-based integral sliding mode control strategy

To pursue the design of Certainty equivalence-based ISMC, the integral sliding manifold [40] is defined as follows

$$\begin{array}{c} s=\dot{e}+c_{2}e+v, \end{array}$$ (47)

where *c*_2_ is a positive constant and *v* is an integral term that results in the elimination of reaching phase. The time derivative of (47) along (20) and (16) produces the following equation.

$$\begin{array}{c} \dot{s}={L_{f}}^{2}h(x)+L_{g}L_{f}h(x)(u+F(x,t))-{\ddot{z}}_{1ref}+c_{2}\dot{e}+\dot{v} \end{array}$$ (48)

Now, using (18) and (19) in (48), we get the following expression.

$$\begin{array}{c} \dot{s}={L_{f}}^{2}h(x)+L_{g}L_{f}h(x)(u+f_{st}\left(x\right)+f_{un}\left(x,t\right))-{\ddot{z}}_{1ref}+c_{2}\dot{e}+\dot{v} \end{array}$$ (49)

The overall control law appears as follows

$$\begin{array}{c} u=u_{o}+u_{eq}+u_{d} \end{array}$$ (50)

Substituting (50) in (49), yields

$$\begin{array}{c} \dot{s}={L_{f}}^{2}h(x)+L_{g}L_{f}h(x)(u_{o}+u_{eq}+u_{d}+f_{st}\left(x\right)+f_{un}\left(x,t\right))-{\ddot{z}}_{1ref}+c_{2}\dot{e}+\dot{v} \end{array}$$ (51)

To calculate the regularizing/equivalent control input, assuming the uncertain terms and $\dot{s}$ equal to zero, which results in

$$\begin{array}{c} u_{eq}=-\frac{{L_{f}}^{2}h(x)}{L_{g}L_{f}h(x)} \end{array}$$ (52)

This selection of *u*_*eq*_ decouples the system and gives desired output for a nominal plant with no faults. Taking $\dot{v}$ as

$$\begin{array}{c} \dot{v}={\ddot{z}}_{1ref}-c_{2}\dot{e} \end{array}$$ (53)

The selection of $\dot{v}$ and *v*(0) = −*s*(0) confirms the elimination of reaching phase and, thus, sliding mode is initiated from the very initial time. In order to cancel the effects of the structured uncertainties, an equivalent cancellation law *u*_*o*_ is proposed as follows

$$\begin{array}{c} u_{o}=-\hat{\Delta }\Psi \left(x\right), \end{array}$$ (54)

where $\hat{\Delta }$ represents the estimated value of the unknown parameter. The discontinuous control law *u*_*d*_ appears as follows

$$\begin{array}{c} u_{d}=-\frac{1}{L_{g}L_{f}h(x)}(M_{1}s+M_{2}sign\left(s\right)), \end{array}$$ (55)

where *M*_1_ and *M*_2_ are positive gains. The overall control law can be written as

$$\begin{array}{c} u=-\hat{\Delta }\Psi \left(x\right)-\frac{1}{L_{g}L_{f}h(x)}({L_{f}}^{2}h(x))-\frac{1}{L_{g}L_{f}h(x)}(M_{1}s+M_{2}sign\left(s\right)) \end{array}$$ (56)

**Fig 2:**
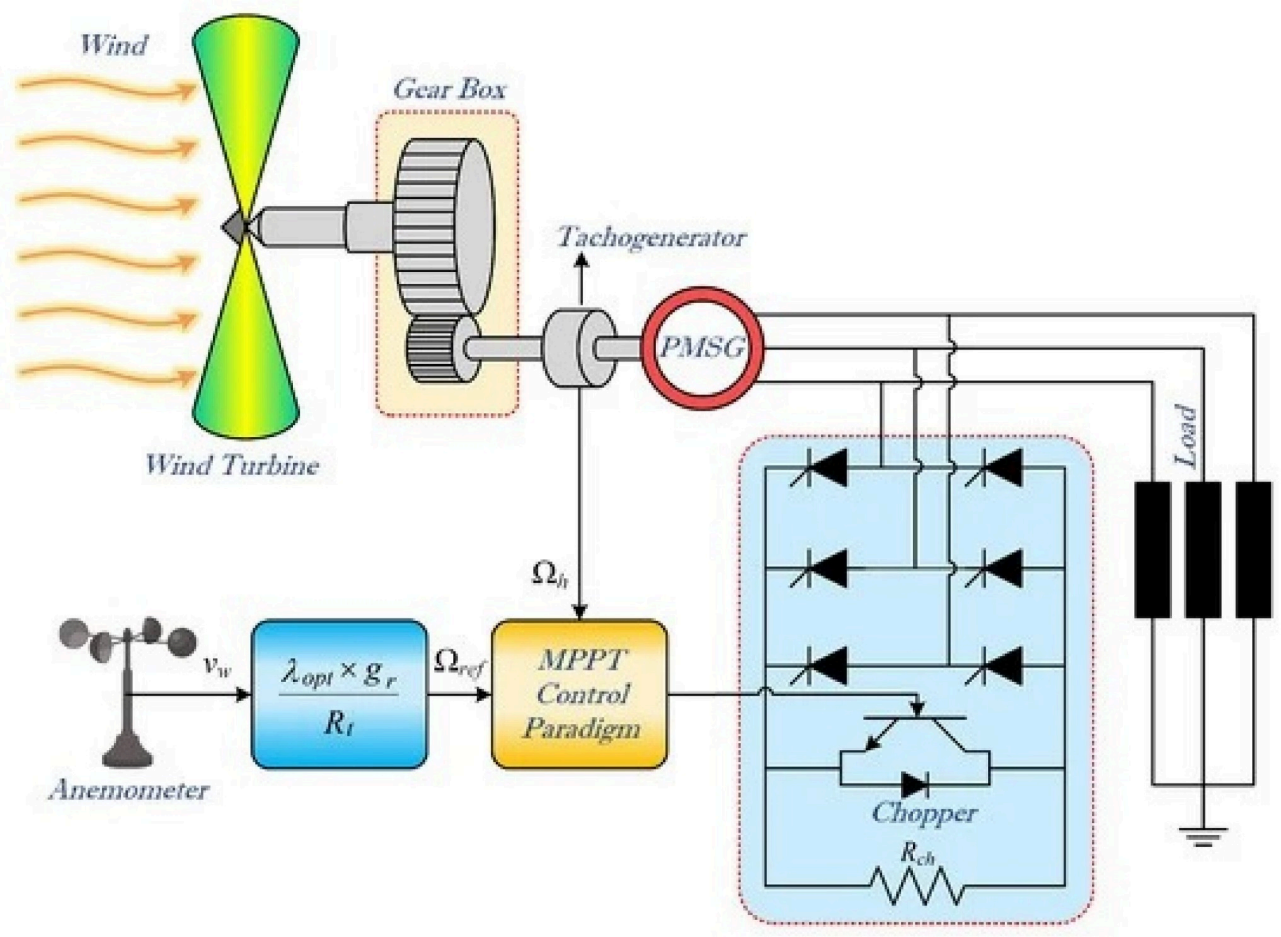
Schematic of the overall system, i.e., PMSG-based WECS.

The final control law 56 enforces the sliding mode 47 along the sliding surface, given in (49), in a finite instant of time. The schematic of the overall closed-loop system, i.e., a variable speed wind turbine (VSWT), a gearbox, power electronic converters and a PMSG coupled with a VSWT, is represented in Fig 2.

## 5 Simulation results and discussion

In this section, the effectiveness of the designed control approaches, proposed for the maximum power extraction, is verified in MATLAB/Simulink. Moreover, the block diagram of the control strategies, in detail is depicted in Fig 2. The assessment, of the proposed control laws, is presented for the following two cases.

Case 1: Stochastic profile of wind speed, operated under constant load, constant inertia and choked input channel
Case 2: Stochastic profile of wind speed, operated under varying load, varying inertia and choked input channel

Note that all the matched disturbances are injected at time *t* ≥ 1 sec. The input channel is choked to 30%, i.e., the input channel is 70% healthy and 30% faulty. The unknown part of the structured fault is 2% which is estimated by adaptation law. The parametric variations for inductance and inertia are carried out at times 5 ≤ *t* ≤ 15 and 30 ≤ *t* ≤ 50.

### 5.1 Case 1

**Fig 3:**
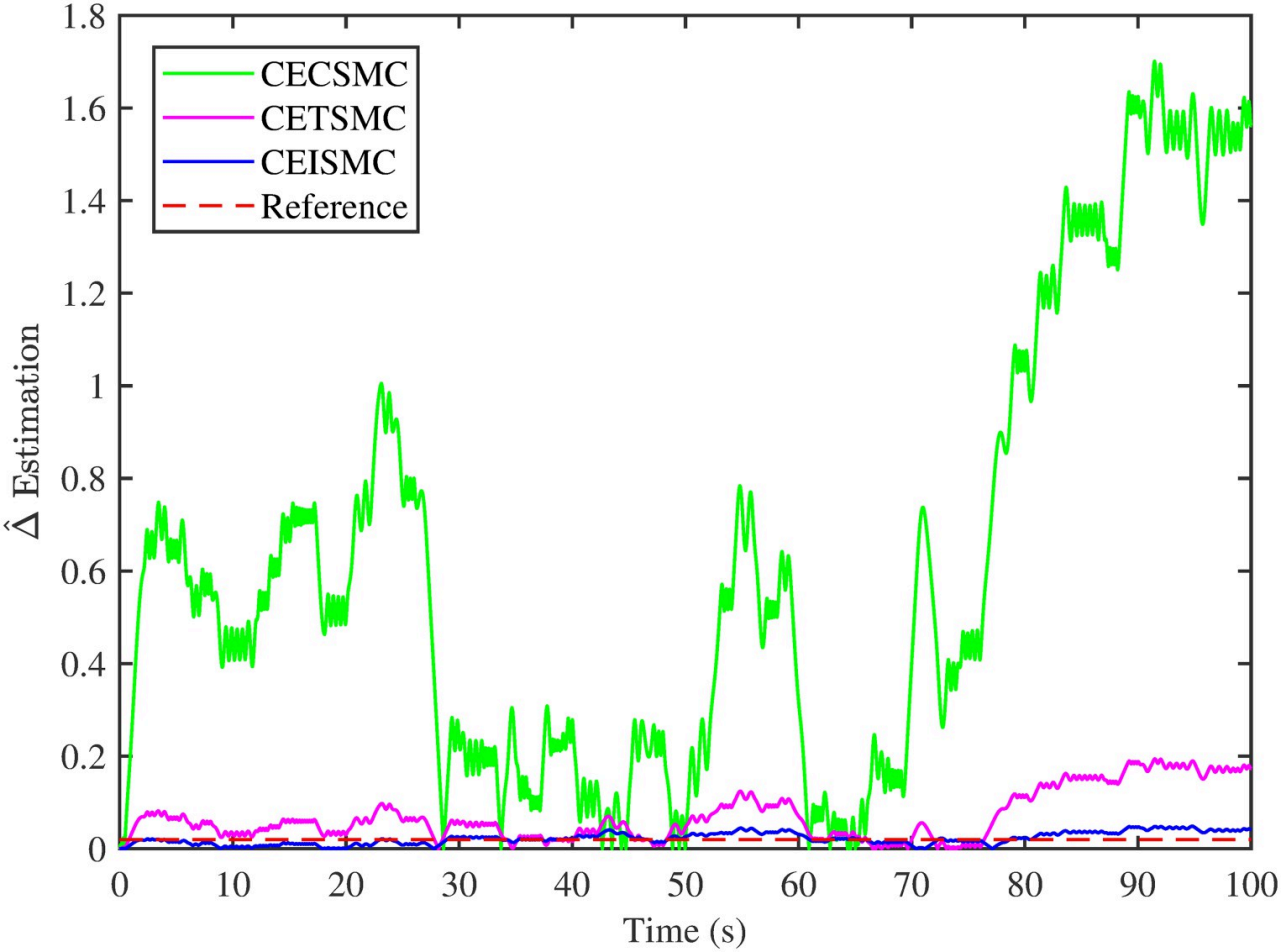
Delta estimation versus time.

**Fig 4:**
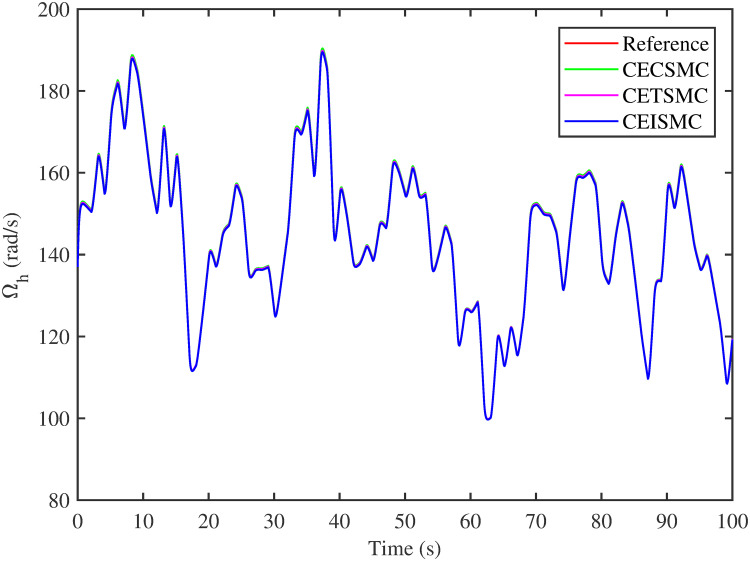
High speed shaft rotational speed.

**Fig 5:**
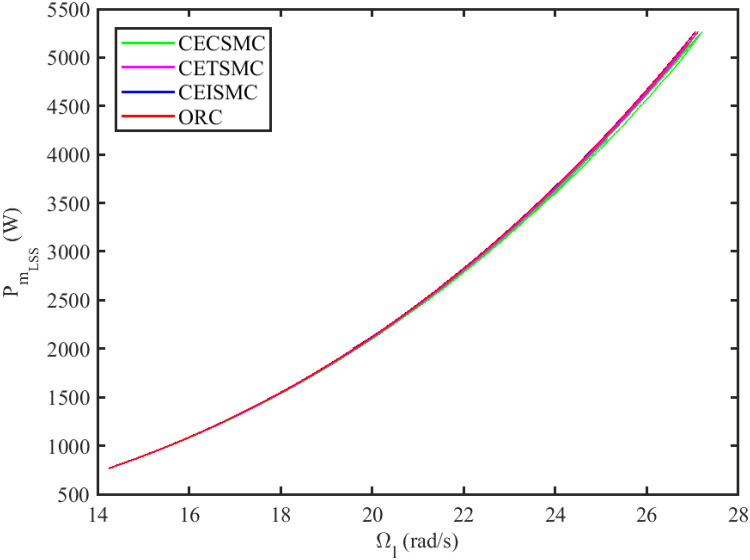
Low-speed shaft rotational speed versus low-speed shaft power.

The simulation results demonstrated in Fig 3 illustrate the estimation of unknown parts, i.e., matched uncertainties, which is subjected to the system via input channel. The aforesaid task is performed by using the proposed control strategies. Fig 3 represents the estimation of the matched uncertainties, corresponding to CEISMC, CETSMC, and CECSMC. It is quite obvious that CEISMC best estimates the unknown uncertainties having known bounds whereas CETSMC also perform quite efficiently but CECSMC possess a steady-state error (SSE), which exists thereafter. Fig 4 depicts the tracking profile achieved by the designed control schemes. The tracking profile is actually the difference between the reference speed of the generator, i.e., Ω_*ref*_, versus the actual speed of the wind turbine, i.e., Ω_*h*_. In Fig 4, It is pretty clear that CEISMC accurately tracks the desired reference speed with a negligible steady-state error. In contrast, the steady-state errors that correspond to CETSMC and CECSMC are quite maximum and show no decline with the passage of time. The Optimal Regime Characteristics (ORC) is basically a combination of maximum power points at different wind speeds. Thus, in Fig 5, a nonlinear relationship between the actual speed of the wind turbine and the power produced by the generator is demonstrated. It is obvious that the characteristics achieved by both CEISMC and CETSMC lie closer to the Optimal Regime Characteristics (ORC) while CECSMC didn’t achieve the optimal results.

**Fig 6:**
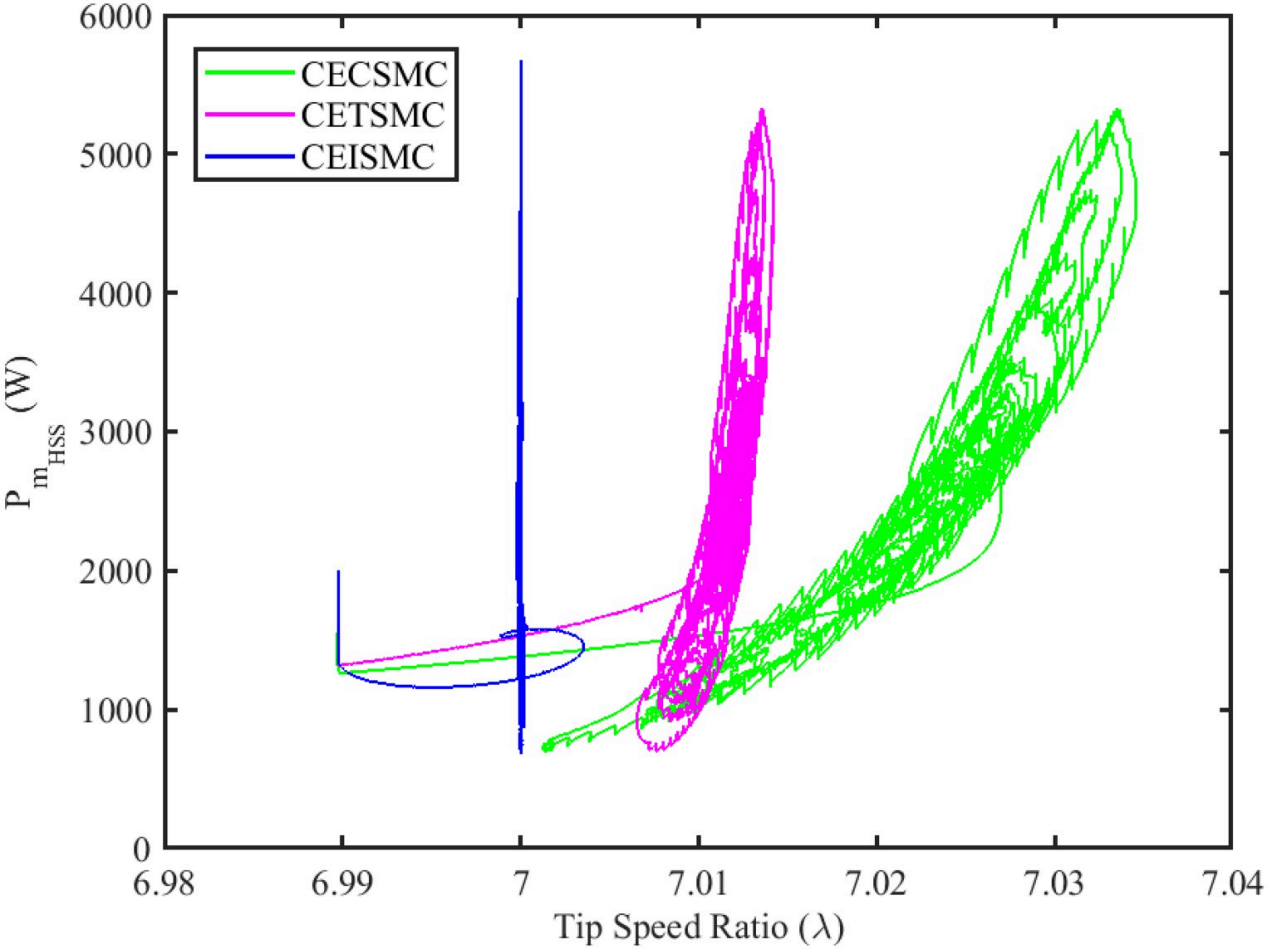
Tip speed ratio versus high speed shaft power.

**Fig 7:**
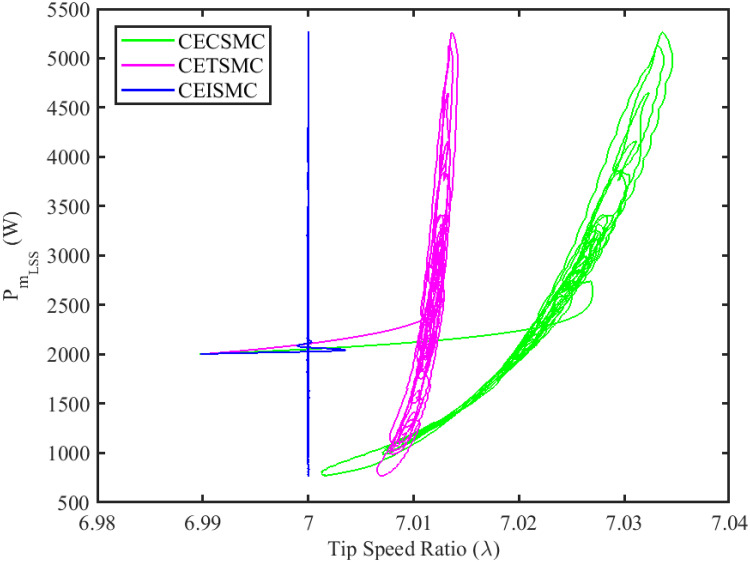
Tip speed ratio versus low speed shaft power.

**Fig 8:**
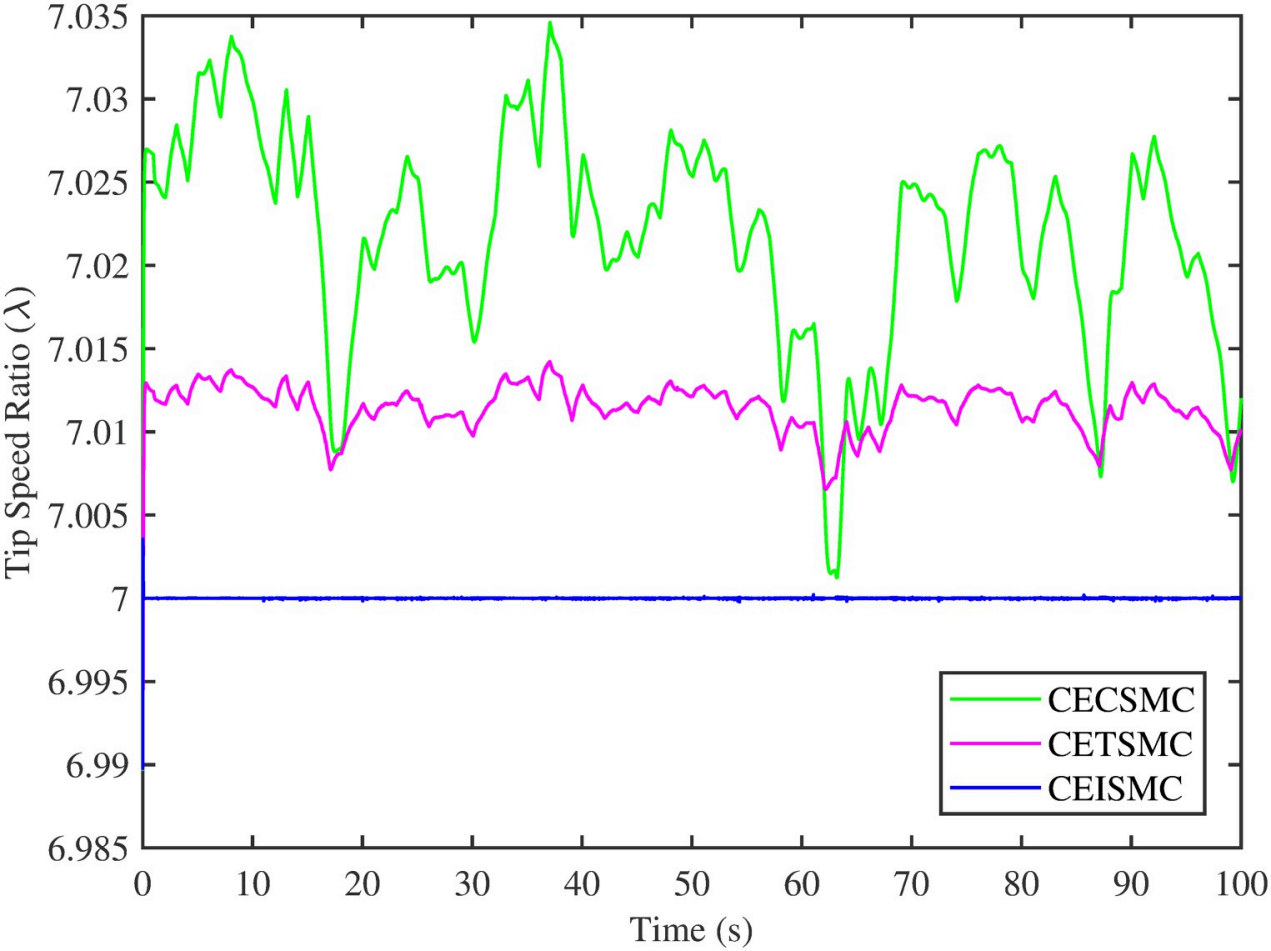
Tip speed ratio versus time.

**Fig 9:**
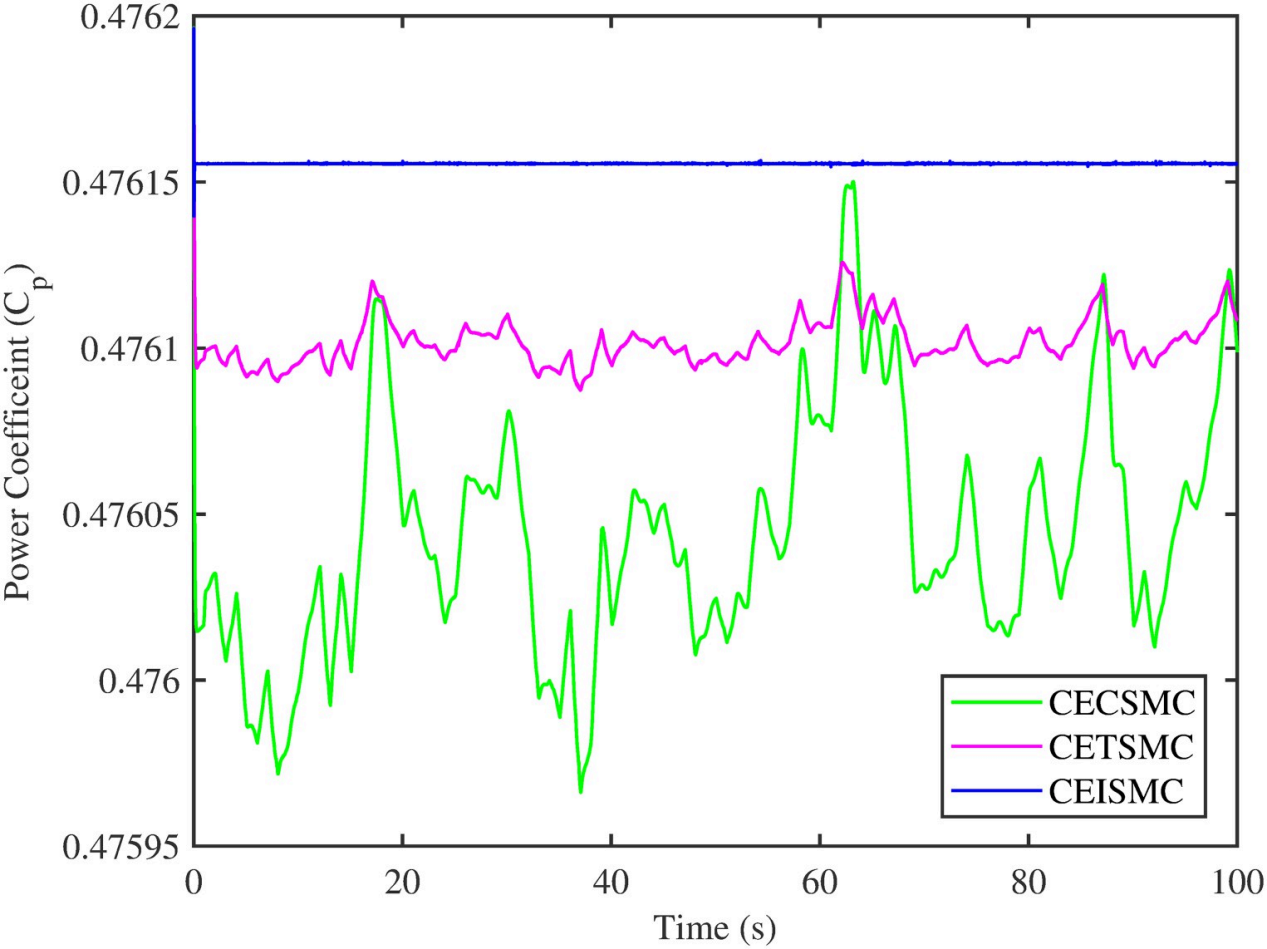
Power coefficient versus time.

In Fig 6, the tip speed ratio that corresponds to a high-speed shaft power is portrayed. It is quite evident that the CEISMC strategy, in contrast to CETSMC and CECSMC, successfully achieved the tip speed ratio, i.e., 7, which is an optimal operating point. In addition, the tip speed ratio at low-speed shaft power, achieved by the designed control schemes, is presented in Fig 7. Again, the CEISMC strategy proved itself as the best candidate to achieve the optimal tip speed ratio at low shaft power. Fig 8 portrays the different tip speed ratio, i.e., λ, versus time that are achieved by the proposed control schemes. In the case of CEISMC scheme, the attained tip speed ratio, i.e., 7, is quite close to the optimal value. However, in CETSMC and CECSMC, it oscillates around 7 and never stabilises itself at any constant position. In Fig 9, the profile of power coefficient *C*_*p*_ that are accomplished by the designed control schemes is demonstrated. The desirable maximum power coefficient, i.e., *C*_*pmax*_, for the VSWT system is 47%, which is quite efficiently attained by CEISMC technique. However, in the case of CETSMC and CECSMC, a little bit variations are observed, which degrades its effectiveness. As the matched faults are injected in the system at *t* ≥ 1, so it is quite obvious in Figs 4–9, that despite the faulty situation there is no degradation observed in any of the aforesaid performance. It means that the designed control strategies has sufficient effectiveness to overcome the uncertain condition and thus, the objective is fulfilled, i.e., MPPT is achieved. In Figs 6–9, it is analysed that the designed control schemes reduced the chattering phenomenon. The CEISMC strategy quite effectively mitigates the chattering phenomena while the CETSMC and CECSMC algorithms possess a little bit of chattering, which is dangerous for the actuator health. All the presented simulations are performed for the system, which possess modelled dynamics along with matched uncertainties. So, it is concluded that the proposed control schemes handled the stochastic nature of wind speed, counteract the matched faults, reduced the chattering effect, and efficiently achieved the MPPT.

### 5.2 Case 2

**Fig 10:**
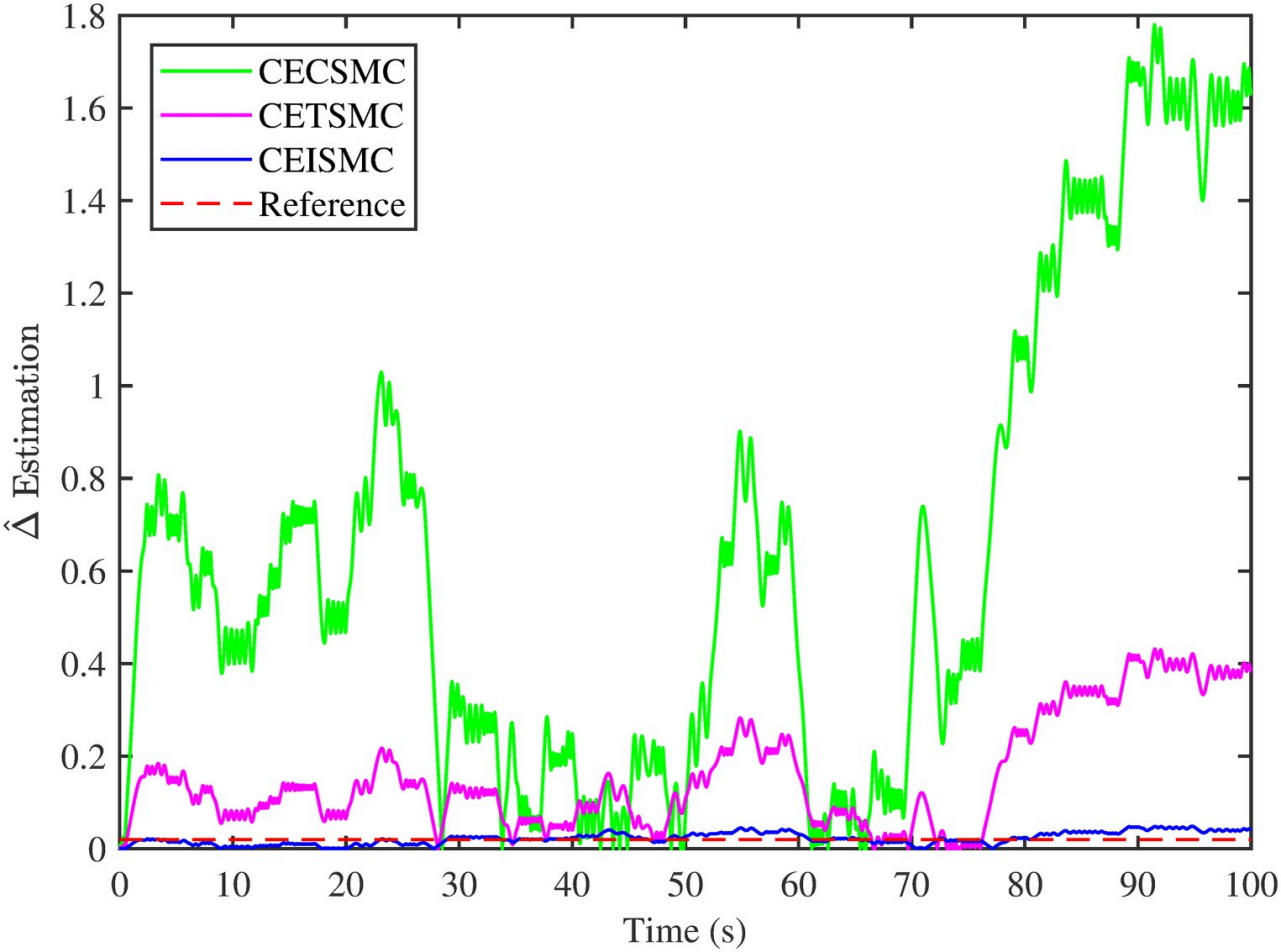
Delta estimation versus time.

**Fig 11:**
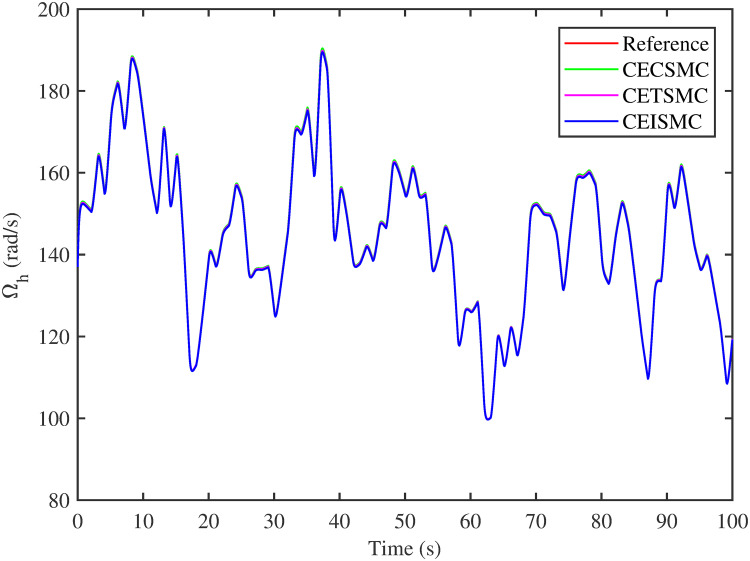
High speed shaft rotational speed.

**Fig 12:**
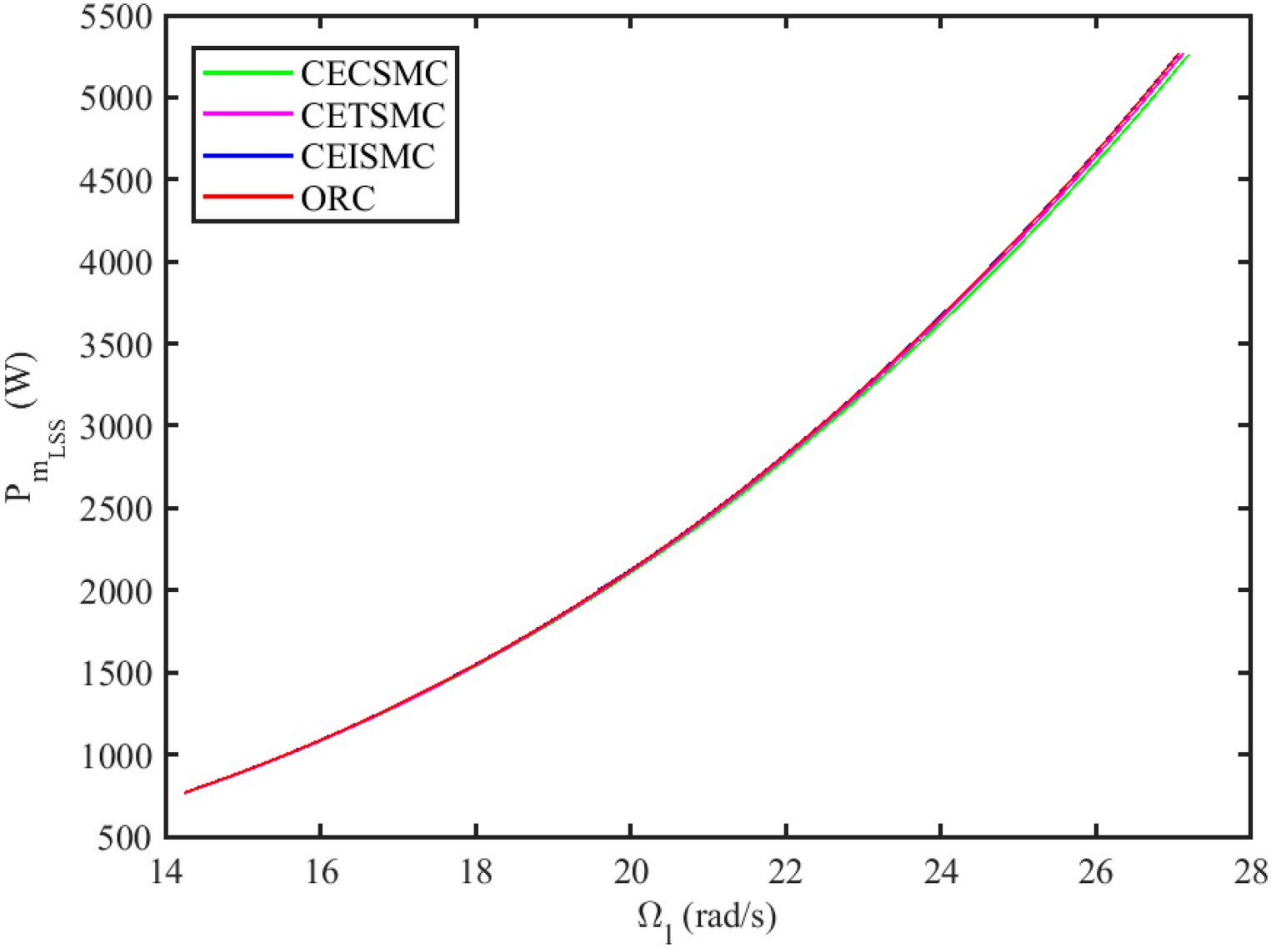
Low shaft rotational speed versus low-speed shaft power.

In this subsection, the efficacy of the designed control strategies for the system that is subjected to un-modelled dynamics, i.e., variable load and inertia, are discussed. The system, which is under consideration, is also exposed to uncertainties that are entering via input channel, i.e., matched uncertainties. In Fig 10, the estimation of matched uncertainties, i.e., $\hat{\Delta }$, by using the designed strategies are illustrated. It is pretty obvious in Fig 10 that the CEISMC scheme accurately tracks the desired reference trajectory and has a negligible steady-state error, i.e., 2%. While the CETSMC quite closely tracks the desired reference trajectory and lies in the vicinity but still has a sufficient amount of tracking error whereas the CECSMC strategy doesn’t track the desired trajectory and possesses the steady-state error, which exists thereafter. In Fig 11, the difference between the actual speed of the generator, i.e., Ω_*ref*_, and the references trajectory, i.e., Ω_*h*_, is depicted. It is quite clear that the CEISMC scheme tracks the reference trajectory in a short time span with a minimum steady-state error. However, the designed strategies, i.e., CEISMC and CETMC, either suffer from long settling time or maximum steady-state error. Fig 12 demonstrates the nonlinear plot comparative analysis between the actual speed of the wind turbine and the power produced by generator. The comparative profiles that are achieved by both CEISMC and CETMC schemes best matches the optimal regime characteristics, which outshine its supremacy in the achievement of MPPT. However, the performance achieved by CECSMC is out of the way and is not suitable for the attainment of an efficient MPPT.

**Fig 13:**
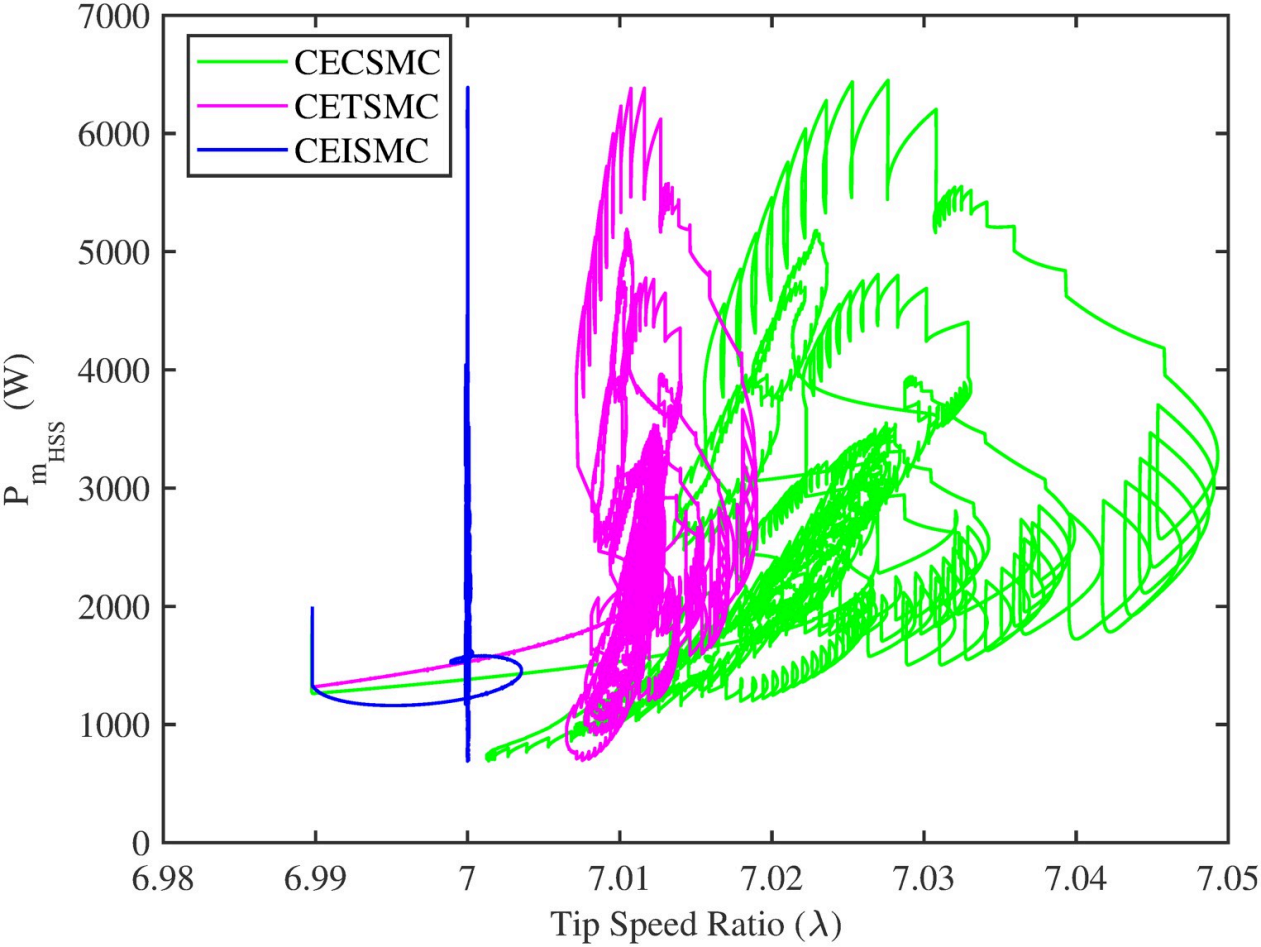
Tip speed ratio versus high speed shaft power.

**Fig 14:**
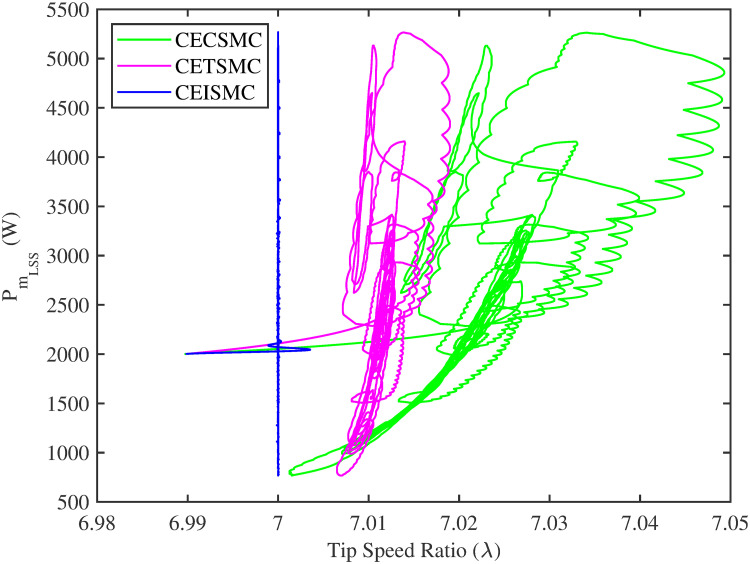
Tip speed ratio versus low speed shaft power.

**Fig 15:**
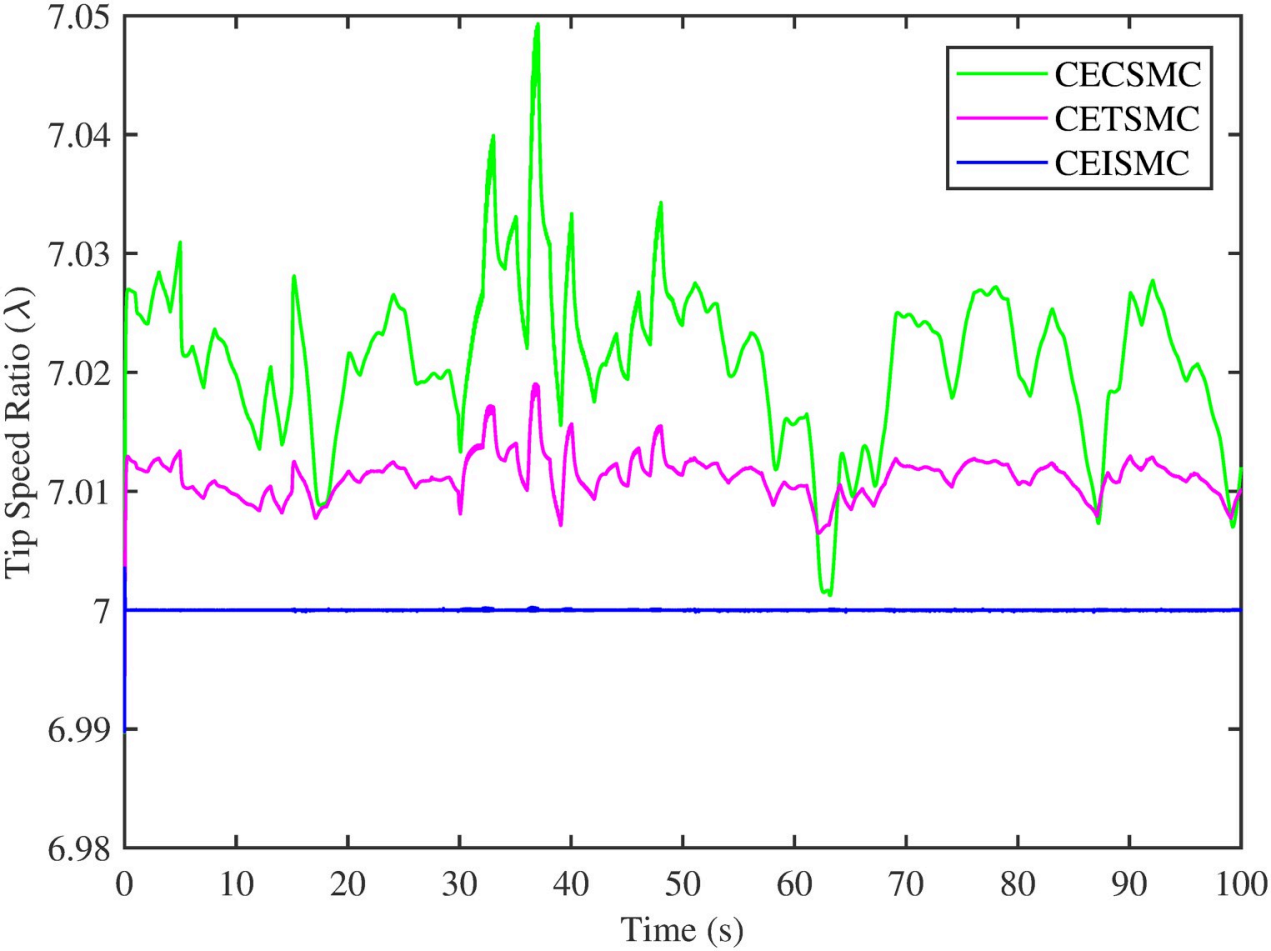
Tip speed ratio versus time.

**Fig 16:**
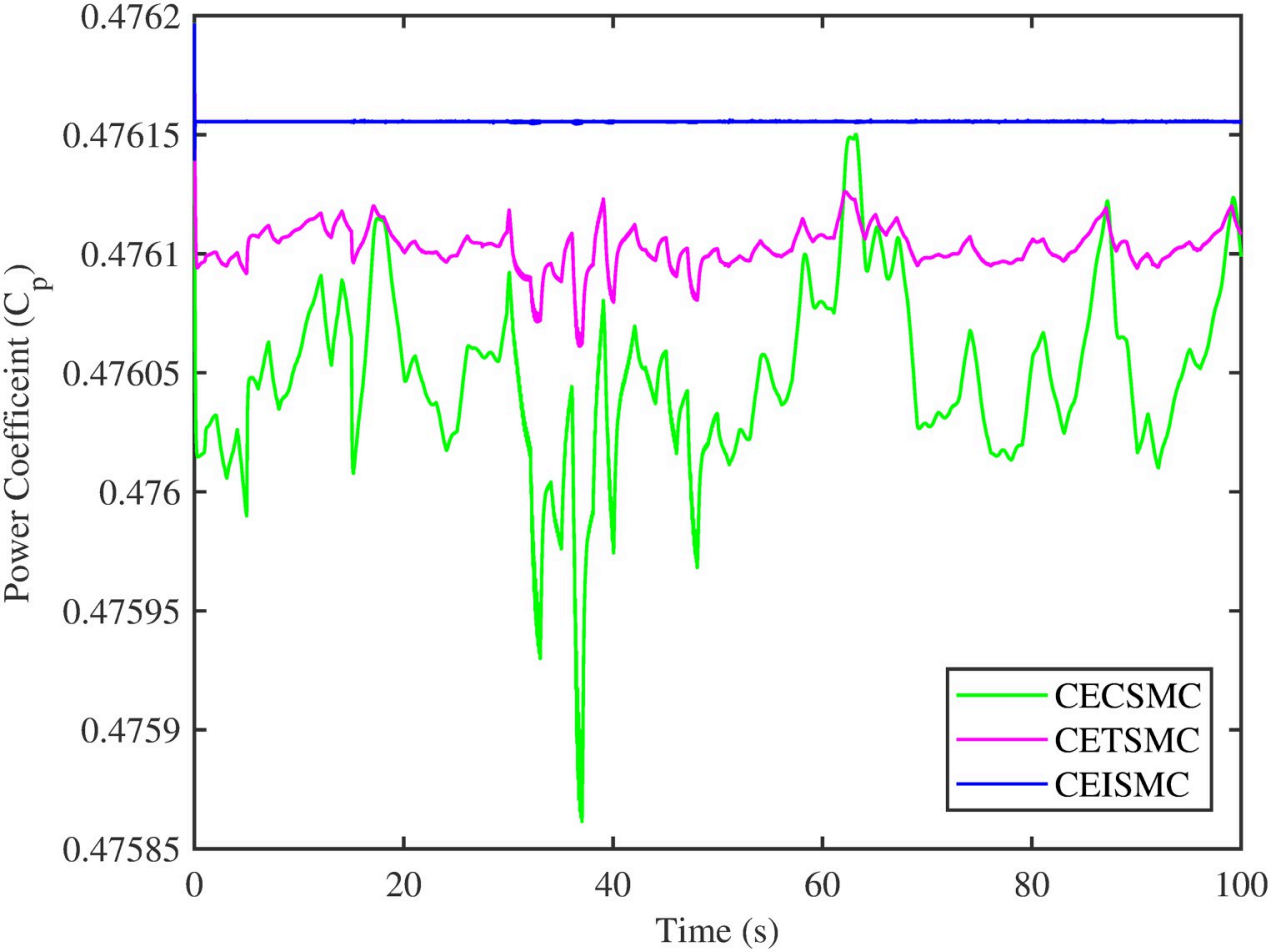
Power coefficient versus time.

Fig 13 shows the comparative illustration of the wind turbine’s power and its tip speed ratio at a high-speed shaft while Fig 14 also represents the same parameters but at a low shaft speed. It is evident in both scenarios that the CEISMC strategy quite efficiently attains the optimal tip speed ratio, i.e., 7, for the stochastic nature of wind speed. However, by employing CETSMC and CECSMC strategies, the tip speed ratio deviates from the optimal value. In Fig 15, the tip speed ratios, i.e., λ, versus time, corresponding to the designed control strategies, are depicted. It is clearly portrayed that the CEISMC scheme accomplishes the optimal tip speed ratio, i.e., 7. while the tip speed ratios that correspond to CETSMC and CECSMC strategies, oscillate around 7 and don’t stabilise at any constant position. Fig 16 depicts the comprehensive profile of the power coefficient *C*_*p*_. It demonstrates the power coefficient through-out the course of the simulation. It can be obviously seen that the value of *C*_*p*_ lie in the close vicinity of *C*_*pmax*_ despite all the fluctuations in the wind speed, un-modelled dynamics and matched faults. The desirable maximum power coefficient *C*_*pmax*_ for the VSWT system is 47%, which is accurately achieved by CEISMC without any oscillations. However, in the case of CETSMC and CECSMC schemes, the undesirable oscillations are observed, which is an unwanted phenomenon. The designed control strategies play a vital role in the mitigation of matched faults. It can be clearly seen in Figs 11–16 that uncertainties are mitigated while ensuring closed-loop stability. Also, the MPPT is achieved. The control schemes, i.e., CETSMC and CECSMC, are a little bit affected by unmodeled dynamics. However, CEISMC remained unaffected and over-perform. So, it is concluded that the proposed control scheme, i.e., CEISMC, is an appealing candidate for the achievement of MPPT in WECS.

## 6 Conclusion

A certainty equivalence-based robust CSMC, TSMC and ISMC have been presented in this work to extract maximum power from a wind energy system, termed PMSG-WECS. The considered system is exposed to both structure and unstructured uncertainties/faults, i.e., load and inertia. Initially, the system, i.e., PMSG-WECS, is converted into controllable canonical form and stability of the zero dynamics is guaranteed. Secondly, certainty equivalence-based robust control laws are designed. The said control strategies efficiently attain the desired performance, i.e., regarding MPPT, for the WECS. The chattering issue, which can affect the actuator’s health, is sufficiently reduced in the control inputs. To comparatively analyse the performance of the designed control techniques, CSMC asymptotically achieves the sliding mode enforcement. As a result, the system output trajectory effectively tracks the desired reference path. While TSMC acquires a finite time error convergence along with suppressed chattering. The aforementioned both control strategies are pretty sensitive to uncertainties in the reaching phase, therefore, an ISMC scheme is proposed. In contrast, an ISMC strategy attains a sliding mode from the initial point, thus eliminating a reaching phase. The absence of reaching phase efficiently improves the robustness of the system to the un-modelled dynamics as well as the sliding mode enforcement is accomplished in a finite time instant. The overall control strategies are numerically developed and stability analysis is guaranteed via the Lyapunov candidate function. The theoretical claims are certified via computer simulations performed in MATLAB/Simulink. The results obtained are discussed thoroughly and it is concluded that ISMC outshines all the reported techniques in the overall performance.
